# Quantitative analysis and stochastic modeling of osteophyte formation and growth process on human vertebrae based on radiographs: a follow-up study

**DOI:** 10.1038/s41598-024-60212-5

**Published:** 2024-04-24

**Authors:** Tong Wu, Changxi Wang, Kang Li

**Affiliations:** 1 West China Biomedical Big Data Center, West China Hospital, Sichuan University, Chengdu, 610041 China; 2 Orthopedics Department of West China Hospital, Sichuan University, Chengdu, 610041 China; 3 Sichuan University - Pittsburgh Institute, Sichuan University, Chengdu, 610207 China

**Keywords:** Radiography, Applied mathematics, Musculoskeletal system, Disease prevention

## Abstract

Osteophytes are frequently observed in elderly people and most commonly appear at the anterior edge of the cervical and lumbar vertebrae body. The anterior osteophytes keep developing and will lead to neck/back pain over time. In clinical practice, the accurate measurement of the anterior osteophyte length and the understanding of the temporal progression of anterior osteophyte growth are of vital importance to clinicians for effective treatment planning. This study proposes a new measuring method using the osteophyte ratio index to quantify anterior osteophyte length based on lateral radiographs. Moreover, we develop a continuous stochastic degradation model with time-related functions to characterize the anterior osteophyte formation and growth process on cervical and lumbar vertebrae over time. Follow-up data of anterior osteophytes up to 9 years are obtained for measurement and model validation. The agreement test indicates excellent reproducibility for our measuring method. The proposed model accurately fits the osteophyte growth paths. The model predicts the mean time to onset of pain and obtained survival function of the degenerative vertebrae. This research opens the door to future quantification and mathematical modeling of the anterior osteophyte growth on human cervical and lumbar vertebrae. The measured follow-up data is shared for future studies.

## Introduction

The spine is a crucial human body component that degenerates over time^1^. One of the most critical indicators of spinal degeneration is osteophyte formation, which is reported to be found in 20–30% of the elderly population and requires proper diagnosis and in-time interventions in clinical practice^2–4^.

Osteophytes most commonly appear at the anterior edges of the cervical and lumbar vertebral bodies^5,6^. They are not symptomatic early but would keep growing as the patient ages^7^. The anterior osteophytes that continuously protrude would result in neck/back pain at the point where they mechanically compress the spinal nerve roots or soft tissue structures^8,9^. In clinical practice, the oversized anterior osteophyte is a significant concern^2,5^. For patients with anterior osteophyte growing on the spine, lateral radiographs are mainly used for osteophyte length evaluation and diagnosis^4^. The accurate measurement of the anterior osteophyte length on lateral radiographs and the understanding of the temporal progression of anterior osteophyte growth are of vital importance to clinicians to develop effective treatment plans.

To date, two categories of measuring methods have been proposed to quantify the anterior osteophyte length on lateral radiographs. The first category^10,11^ records the measured data directly without processing while the second category^12^ uses an index to represent the osteophyte length. Regression models are mainly used to characterize the osteophyte growth process. Watanabe et al.^13^ use a linear regression model to describe the relationship between the osteophyte index and age. Gender^14^, weight and BMI^15^, bone mass^16^, etc. are found to be the affecting factors of the osteophyte growth process.

The previous studies contribute to the understanding of the biology of osteophyte growth and may facilitate clinical prevention. However, they have the following three limitations. Firstly, existing methods of measuring anterior osteophyte length are not fully applicable in clinical practices since (i) the measurement steps are not well-defined and (ii) the proposed indicator cannot be applied to radiographs with different scale plates. Secondly, the previously used regression models fail to predict the time-related progression of the osteophyte growth process and the time to onset of pain (TTOOP) under uncertainties. Thirdly, to date, most of the studies on osteophytes are cross-sectional, where data is collected only once for each patient. Few longitudinal studies are available for understanding the temporal progression of osteophyte growth over an extended period.

The osteophyte formation and growth process is subject to uncertainties and may undergo significant variations. The specific degeneration initiation time is not a constant value but rather follows a distribution as it differs among the population due to factors such as incorrect posture and acute spinal injuries^17^. In addition, uncertainties exist during the osteophyte growth process since the biological effects may affect the growth rate of the osteophyte^18^ and there are person-to-person variations due to their genetic and lifestyle differences^19^. Compared to the commonly used regression model, stochastic models that incorporate the temporal uncertainties and random factors are suitable for capturing the evolution of osteophyte formation and growth over time^20^.

Currently, a variety of stochastic models have been implemented in degradation analysis^21^. The survival function and the TTOOP can be obtained and the corresponding condition-based maintenance plans are scheduled for the systems subject to degradation to reduce the pain risk^22^. Despite their straightforward physical interpretations and tractable mathematical properties, the applications of stochastic models in characterizing the human spine degeneration process are still at their early stages.

In this study, a robust measuring method for anterior osteophyte length on lateral radiographs is proposed, which can be applied to spine degeneration quantification in clinical studies. Furthermore, we develop a stochastic model to characterize and predict the temporal progression of osteophyte formation and growth with high accuracy, validity and interpretability. Considering that the actual lifetime data are usually censored and aperiodic, the maximum likelihood estimation (MLE) method is used to estimate the parameters. The model is validated using long-term follow-up data. This work contributes not only to the understanding of the osteophyte growth process, but also to the survival assessment and prognostic care for degenerative spinal vertebrae in clinical practice.

## Results

### Agreement of measurements

In this study, we use the osteophyte ratio index (ORI) to quantify the anterior osteophyte length on lateral radiographs. A robust measuring method is proposed as shown in Fig. 7b legend. Four observers with different ages and experiences are included in the agreement tests to compare the robustness of the measuring methods proposed in this study and Walraevens et al.^12^’s study. The results of agreement tests for the two measuring methods are shown in Table 1.

**Table 1:** Agreement of measurements.

			ORI^c^ with well-defined measuring steps proposed in this study	Index in Walraevens et al.^12^’s study
Intra-observer	Observer I	ICC^a^	0.962 (95% CI: 0.933–0.978)	0.221 (95% CI: 0.00–0.501)
		PD^b^	5.5%	29.4%
	Observer II	ICC^a^	0.960 (95% CI: 0.929–0.978)	0.640 (95% CI: 0.440–0.779)
		PD^b^	4.5%	18.6%
	Observer III	ICC^a^	0.989 (95% CI: 0.980–0.994)	0.742 (95% CI: 0.568–0.850)
		PD^b^	3.3%	15.2%
	Observer IV	ICC^a^	0.968 (95% CI: 0.938–0.983)	0.751 (95% CI: 0.599–0.851)
		PD^b^	5.0%	13.6%
Inter-observer	Observer I and II	ICC^a^	0.971 (95% CI: 0.947–0.984)	0.219 (95% CI: 0.00–0.453)
		PD^b^	4.6%	20.9%
	Observer I and III	ICC^a^	0.970 (95% CI: 0.941–0.984)	0.388 (95% CI: 0.110–0.606)
		PD^b^	4.8%	16.8%
	Observer I and IV	ICC^a^	0.948 (95% CI: 0.910–0.970)	0.332 (95% CI: 0.00–0.588)
		PD^b^	6.9%	20.7%
	Observer II and III	ICC^a^	0.945 (95% CI: 0.906–0.969)	0.632 (95% CI: 0.433–0.772)
		PD^b^	6.6%	21.1%
	Observer II and IV	ICC^a^	0.980 (95% CI: 0.965–0.989)	0.741 (95% CI: 0.584–0.844)
		PD^b^	4.0%	18.2%
	Observer III and IV	ICC^a^	0.914 (95% CI: 0.853–0.950)	0.636 (95% CI: 0.420–0.781)
		PD^b^	9.0%	15.9%

^a^Intra-class correlation coefficient (ICC)^23^: above 0.90—excellent; between 0.75 and 0.90—good; between 0.50 and 0.75—moderate; below 0.50—poor.

^b^Percentage difference (PD) =|difference between two values/the baseline value| *100%^24^: a smaller PD value indicates a lower difference and thus higher agreement.

^c^Osteophyte ratio index (ORI) = length of the osteophyte/width of the vertebra body under the proposed measuring steps.

### Validation of the osteophyte formation and growth process model

**Figure 1:**
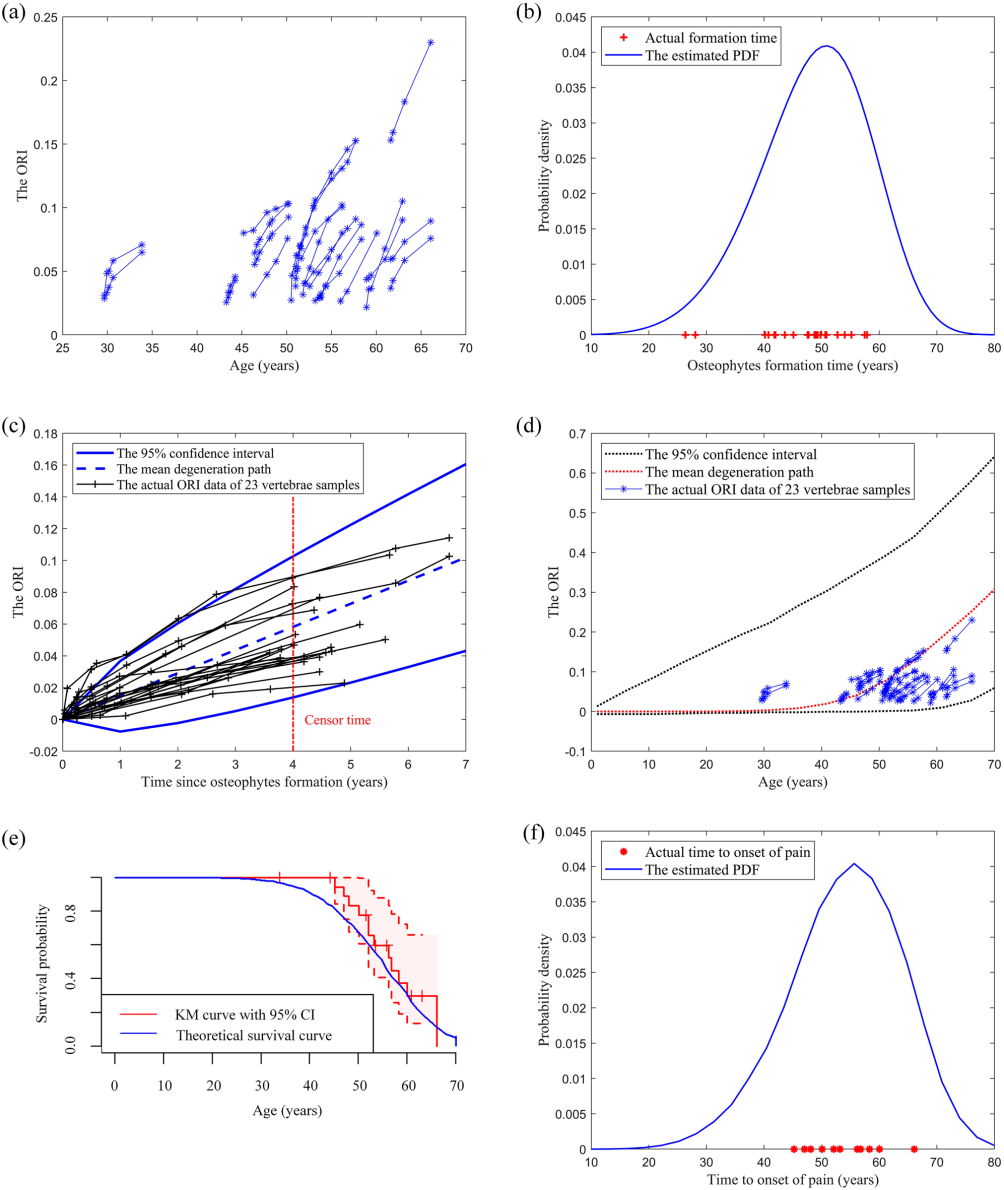
(**a**) The osteophyte growth data of the 23 cervical vertebrae. ORI refers to the Osteophyte Ratio Index. (**b**) The validation of the Weibull distribution. The actual cervical osteophyte formation time is compared with the estimated probability density function (PDF). (**c**) The validation of the Wiener process. The actual cervical osteophyte growth paths are compared with the predicted confidence interval (CI). (**d**) The actual and predicted osteophyte formation and growth process of a cervical vertebra. (**e**) The comparison of the theoretical cervical vertebra’s survival function and the Kaplan–Meier (KM) curve of the actual data. (**f**) The comparison of the cervical vertebra’s time to onset of pain and the actual data with the estimated PDF.

**Figure 2:**
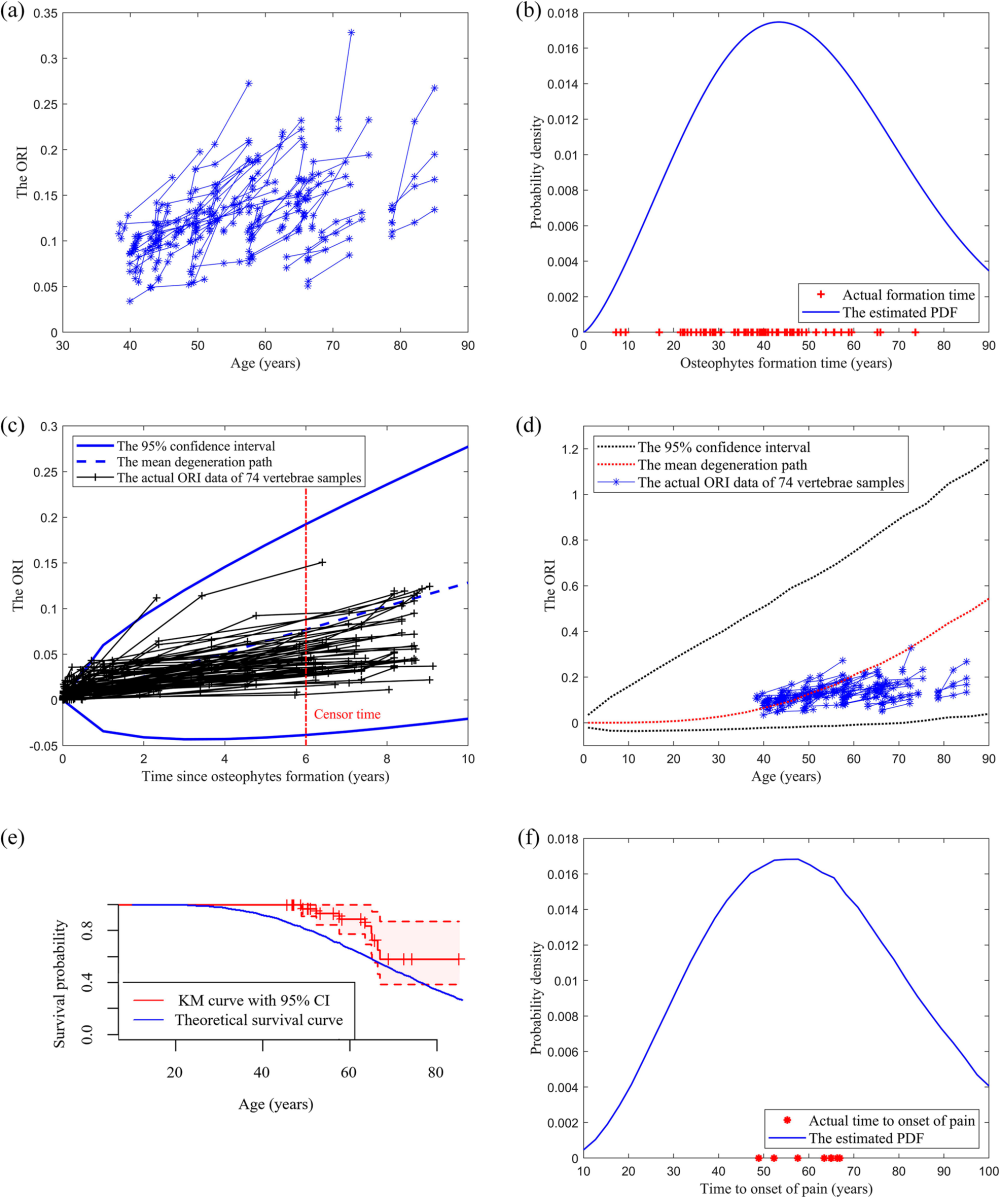
(**a**) The osteophyte growth data of the 74 lumbar vertebrae. ORI refers to the Osteophyte Ratio Index. (**b**) The validation of the Weibull distribution. The actual lumbar osteophyte formation time is compared with the estimated probability density function (PDF). (**c**) The validation of the Wiener process. The actual lumbar osteophyte growth paths are compared with the predicted confidence interval (CI). (**d**) The actual and predicted osteophyte formation and growth process of a lumbar vertebra. (**e**) The comparison of the theoretical lumbar vertebra’s survival function and the Kaplan–Meier (KM) curve of the actual data. (**f**) The comparison of the lumbar vertebra’s time to onset of pain and the actual data with the estimated PDF.

The results of ORI measurements of the 23 cervical vertebrae samples and 74 lumbar vertebrae samples are shown in Figs. 1a and 2a respectively, where the x-axis represents the ages of patients, and the y-axis represents the corresponding ORIs. Based on the MLE functions described in Eq. (6), the parameters of the Weibull distribution are estimated as $\left\{ {\hat{a} = {52}{\text{.5216}}, \, \hat{b} = {5}{\text{.7469}}} \right\}$ and $\left\{ {\hat{a} = {5}4.9779, \, \hat{b} = {2}{\text{.3443}}} \right\}$ for cervical and lumbar vertebrae respectively. The actual osteophytes formation time of samples and the probability density function (PDF) of the fitted Weibull distribution for cervical and lumbar vertebrae are shown in Figs. 1b and 2b respectively.

Based on MLE functions described in Eq. (7), the parameters of the Wiener process are estimated as $\left\{ {\hat{\mu } = {0}{\text{.0145553}}, \, \hat{\sigma } = {0}{\text{.0113259}}} \right\}$ and $\left\{ {\hat{\mu } = {0}{\text{.012836}}, \, \hat{\sigma } = {0}{\text{.024040}}} \right\}$ for cervical and lumbar vertebrae respectively. Based on the parameters, the 95% confidence interval and the mean path of the predicted ORI of cervical and lumbar vertebrae are shown in Figs. 1c and 2c respectively, where the x-axis represents the duration since the osteophytes are formed, and the y-axis represents the corresponding ORIs.

Based on Eq. (2) and the estimated parameters, the mean and 95% confidence interval of the osteophyte length are obtained and shown in Figs. 1d and 2d for cervical and lumbar vertebrae respectively. The degeneration data are plotted in blue lines with asterisk markers.

### Validation of the survival metrics

To validate the proposed survival metrics, we collected the X-ray image series datasets obtained at discrete time instants during the patients’ follow-up visits, where the ORI value of 0.08 is of the highest count over the 23 cervical vertebrae samples’ ORI series and the value of 0.15 is of the highest count over the 74 lumbar vertebrae samples’ ORI series. For illustration purposes, we assume that the pain threshold values are 0.08 and 0.15, which also conforms to the principles of statistics. The ages when the ORIs reach the pain thresholds are treated as the actual TTOOP values. Compared to other ORI values which only have one or two samples, the counts of 0.08 and 0.15 are higher and thus are more suitable to be used as the pain threshold values. Therefore, without loss of generality, we assume pain thresholds of 0.08 and 0.15 for cervical and lumbar vertebrae respectively to validate the survival metrics.

The theoretical survival curve of cervical/lumbar vertebrae under the pain threshold (i.e., the maximum clinically-acceptable osteophyte size) of 0.08/0.15 is obtained based on Eq. (4) and shown in Figs. 1e and 2e by a blue line. As this study involves survival data, Kaplan–Meier (KM) survival analysis is conducted and the corresponding KM curve of cervical/lumbar vertebrae is shown in Figs. 1e and 2e by a red line, which reflects the actual survival probability of cervical/lumbar vertebrae. The shaded pink region represents the 95% confidence interval of the KM curve.

The PDF of the TTOOP of cervical/lumbar vertebrae under the pain threshold of 0.08/0.15 is estimated based on Eq. (3) and shown in Figs. 1f and 2f by a blue line. The actual TTOOP is shown in Figs. 1f and 2f with red asterisk markers.

Based on Eq. (5), the mean time to onset of pain (MTTOOP) of the cervical/lumbar vertebra given the pain threshold of 0.08/0.15 are obtained. The theoretical and actual MTTOOP for cervical vertebra are 54.11 and 53.76 years respectively (percentage difference = 0.65%). The theoretical and actual MTTOOP for the lumbar vertebra are 60.40 and 60.66 years respectively (percentage difference = 0.43%).

## Discussion

Figures 1b and 2b indicate that the Weibull distribution can accurately characterize the osteophyte formation time of a cervical and lumbar vertebra. Note that all of the test ORI data of each cervical and lumbar vertebra after the censor time (i.e., the data series on the right of the red line) fall into the predicted confidence intervals in Figs. 1c and 2c. It shows that the Wiener process can accurately characterize the osteophyte growth of a cervical and lumbar vertebra. In Figs. 1d and 2d, all the actual data fall into the 95% CI. The large CI is due to the large variation and uncertainties of the osteophyte formation time and growth process among the population. It can increase the likelihood that the interval contains the mean response and also contributes to a more accurate and general prediction of the time to onset of pain for the population with large variations in degeneration performance. Figures 1e and 2e show that the theoretical survival curve derived from the proposed model accurately matches the actual survival curve under the pain-threshold assumption. It is observed from Figs. 1f and 2f that the distribution of the TTOOP obtained from the proposed model accurately characterizes the actual cases. In addition, the theoretical MTTOOP calculated from the proposed model fits the actual data precisely (i.e., The theoretical and actual MTTOOP for cervical vertebra are 54.11 and 53.76 years respectively (percentage difference = 0.65%); The theoretical and actual MTTOOP for the lumbar vertebra are 60.40 and 60.66 years respectively (percentage difference = 0.43%)). Results show that the model is of high accuracy, validity and interpretability.

According to Walraevens et al.^12^, there are no detailed guiding principles for finding the middle point on the irregularly shaped vertebra. As the measurements of osteophyte length are small in magnitude and highly sensitive to noises, measuring methods with ill-defined instructions may result in inaccurate conclusions and low repeatability in clinical practice. Thus, it is urgent to propose a novel robust osteophyte length indicator. In this paper, an indicator ORI (Eq. (1)) with well-defined measuring steps (Fig. 7b legend) is proposed for the quantification of anterior osteophyte length based on lateral radiographs. In the study of Walraevens et al.^12^, AB and CD (Fig. 7c) are measured at the middle of the vertebral body to represent the anteroposterior diameter. However, as the anterior and posterior vertebra contour is curved, the determination of midpoints A/B/C/D is subjective. By comparison, parallel and tangent lines are more objective and suitable for measuring curved structures. In addition, since $h_{0}$ is generally considered as unchanged during the spine degeneration process, the ratio function (Eq. (1)) that divides the $h_{1}$ by $h_{0}$ in the same radiographic image can overcome the scale error caused by various generations of medical equipment and be applied to radiographs of different sizes to reflect the osteophyte size. Table 1 shows that the indicator ORI under the proposed well-defined measuring steps has excellent reliability (i.e., with ICC > 0.90 and low PD values). By comparison, the measurement in Walraevens*,* et al.^12^’s study has lower ICC scores and higher PD values. This indicates that our method is more robust and can be reliably used in clinical practice and in related research to quantify the anterior osteophyte length.

**Figure 3:**
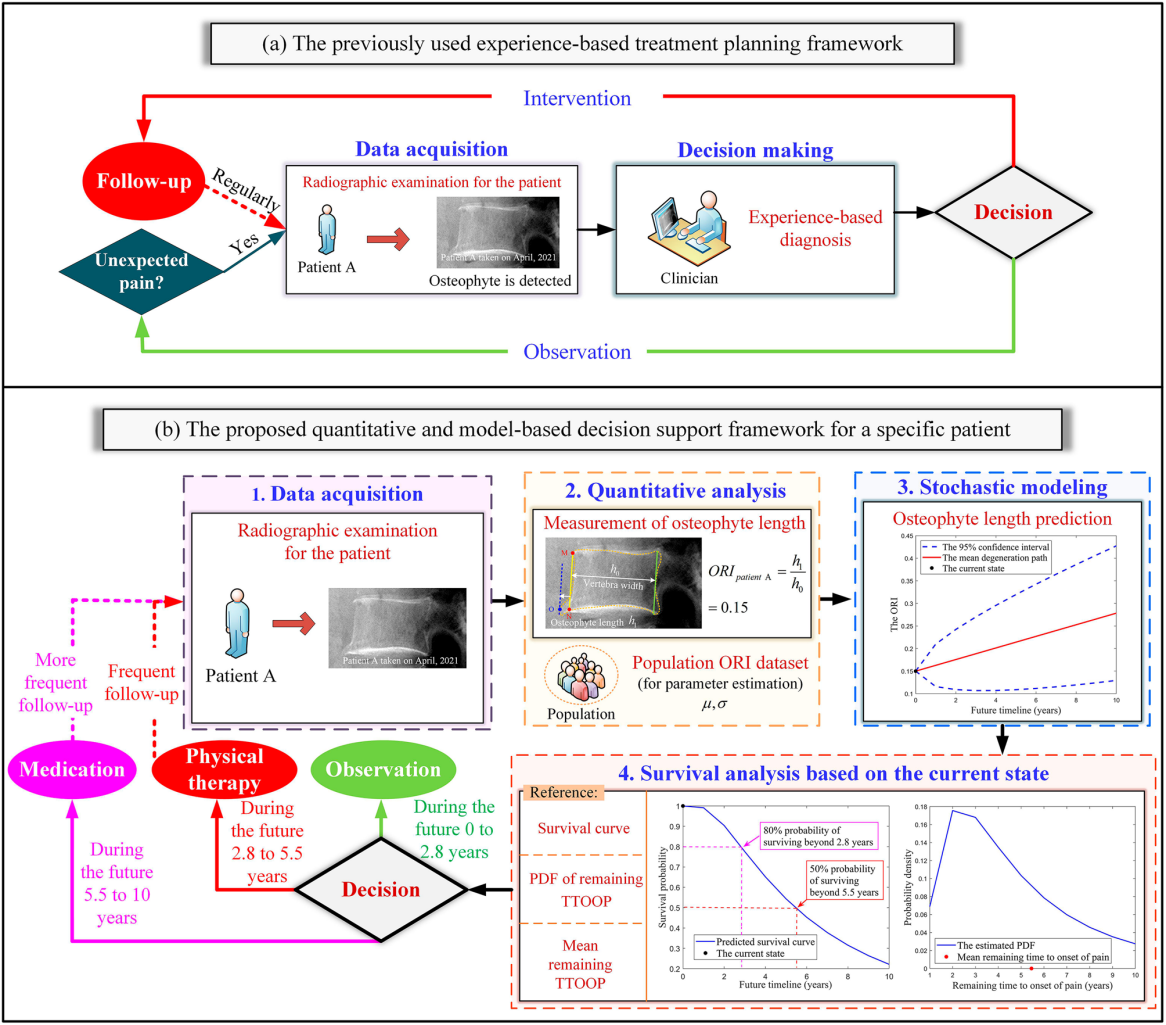
(**a**) The existing experienced-based treatment planning framework. (**b**) The proposed quantitative and model-based decision support framework.

Without an osteophyte growth prediction model for clinical reference, the current treatment plans for degenerative vertebrae are mainly made by clinicians and the treatment quality highly depends on their experience as shown in Fig. 3a ^9^. The treatment plans made by inexperienced clinicians may fail to provide interventions in time and result in unexpected pain. Studies reported that progressive symptoms caused by anterior osteophytes are easily missed during the early evaluation^25^. When severe and unexpected pain develops due to untimely treatment, surgery is required to remove the osteophyte^26^. The development of accurate and valid mathematical models of osteophyte growth is urgently needed to estimate the osteophyte formation time and growth rate, which can be used as a reference for clinical management in the early stage. In this paper, we develop a stochastic model to characterize and predict the temporal progression of osteophyte formation and growth under uncertainties. Compared to the existing stochastic models such as the Wiener processes^21^, Markov Chains^27^, Gamma processes^28^ and Inverse Gaussian processes^29^, we characterize the initiation time of the osteophyte formation by Weibull distribution and incorporate it into the stochastic model by convolution. The proposed model with estimated parameters is especially suitable for characterizing osteophyte formation and growth. Previous studies mainly discuss the prevalence and symptoms of spinal osteophytes rather than quantitative prediction models that would assist prophylactic treatments. In this study, survival metrics can be derived from the stochastic model^20^. As the mean survival time and the pain risk of diseases are major concerns in clinical practice^30^, the derived survival metrics such as MTTOOP and survival function of the degenerated vertebrae can assist clinical decision-making. This study opens the door to the future application of stochastic models to predict degenerative changes in the human spine.

Our work has implications for clinical practices. As the proposed models incorporate temporal uncertainties, random factors and person-to-person variation that evolve in the osteophyte formation and growth process, they can provide population-level inferences for insurance companies to develop proper insurance policies accordingly for different populations. In addition, we propose a quantitative and model-based decision support framework with four steps as shown in Fig. 3b. Step 1: patient A takes a radiographic examination and an osteophyte is detected. Step 2: the indicator ORI is measured to quantify the osteophyte length. Here we assume that the osteophyte is on patient A’s lumbar vertebra and its ORI value is measured to be 0.15 at that time. Step 3: based on the parameters estimated from the population data and the measured initial ORI value, the clinicians can predict how the mean and 95% CI of the patient’s ORI would progress in the future 10 years. Step 4: survival analysis is performed where the survival curve, the PDF plot of the remaining TTOOP and the mean value of the remaining TTOOP are available for the clinician’s reference. For instance, we assume that 80% and 50% are survival probability thresholds for treatment change. The threshold values can be modified by medical professionals. As the survival curve shows that there is an 80% probability of surviving beyond 2.8 years, clinicians can put patient A in observation during the future 0 to 2.8 years. Since the survival probability will decrease to 50% at 5.5 years, frequent follow-up and physical therapy can be scheduled for patient A during the future 2.8–5.5 years. More frequent Follow-up care and medication need to be scheduled during the future 5.5–10 years where the survival probability is under 50%. Note that the treatment plans can be modified in the next follow-up visit based on the patient’s remeasured ORI and the corresponding survival-analysis plots. Compared to the experience-based treatment shown in Fig. 3a, the proposed quantitative and model-based framework in Fig. 3b can provide quantitative prediction for clinicians to make more personalized treatment plans. The survival-analysis plots in Fig. 3b are obtained under the assumed lumbar pain threshold of 0.22. In clinical practice, the pain threshold can be determined based on the criteria provided in the Pain Threshold Definition subsection or modified by the medical professionals. The corresponding survival-analysis plots can be obtained by running the codes in Supplementary B on the MATLAB Platform.

**Figure 4:**
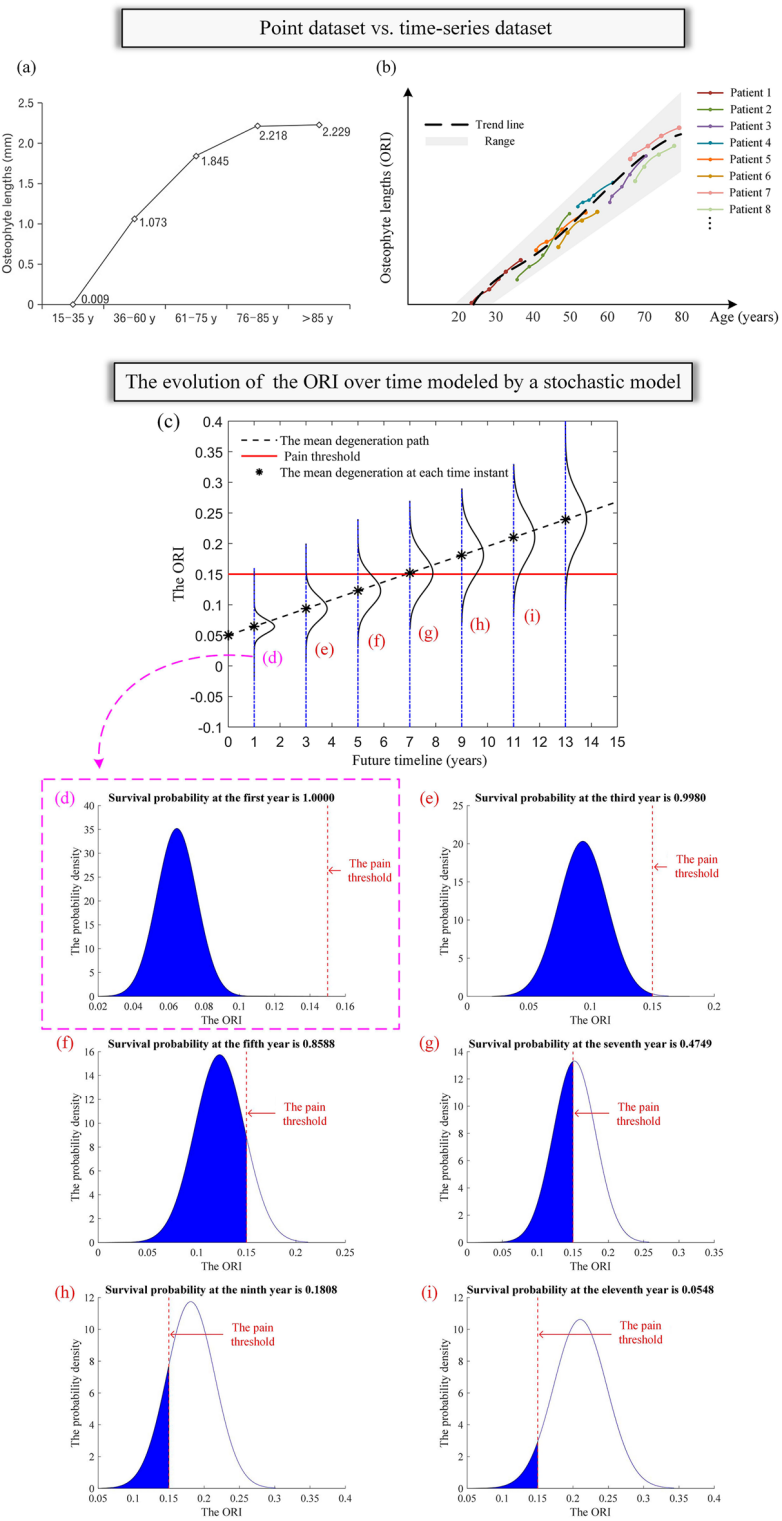
(**a**) Point dataset in Chanapa et al.^8^’s study. (**b**) Time-series dataset in our study. (**c**) The evolution of the PDFs of the ORI over time, assuming that the ORI at the current state is 0.05 and the pain threshold is 0.15 for illustration purposes. Survival probabilities of the vertebra at the first, third, fifth, seventh, ninth and eleventh year are shown in Figures (**d**), (**e**), (**f**), (**g**), (**h**) and (**i**), respectively.

Previous studies are mainly cross-sectional in design and use point datasets to investigate the correlation of osteophyte length with age. For instance, in Chanapa et al.^8^’s study, five age groups (15–35, 36–60, 61–75, 76–85, and > 85 years old) are used and the mean osteophyte length on the vertebral body of patients in each group is obtained as shown in Fig. 4a. There is only one data point for each patient that reflects the osteophyte growth level in that group. However, the progressive changes in osteophyte length of the same patient’s vertebrae are unknown. In the study of^31,32^, although 2–3 years of follow-up data are collected, they are rather short periods considering the slow and progressive osteophyte growth process. As a result, little change in osteophyte growth is observed during the short periods of the studies^33^. With discrete-time datasets or short-term follow-up data, there is a lack of historical data for model fitting and validation. Long-term follow-up data are crucial for understanding osteophyte growth and developing predictive models. In this study, we obtained long-term time-series follow-up radiographs as illustrated in Fig. 4b. To the best of the authors' knowledge, it is relatively new to apply time-series osteophyte data for the progression assessment of osteophyte growth. It helps to understand the temporal progression of osteophyte growth over an extended period and to develop models for osteophyte growth prediction.

In previous studies, deterministic models such as linear and logistic regression models are typically used in clinical analysis to predict the development of osteophyte-related diseases^34–37^. Compared to that, we used the stochastic process model which provides a range of estimates rather than point estimates. The range of estimates that, each of which is associated with a probability, can characterize the randomness and the temporal uncertainties associated with the evolution of the degeneration process. The uncertainties originate from both observable and unobservable factors including the variability of patients, the differences in medicine interventions, etc. The model uses variables to incorporate the random effects of those factors on the osteophyte growth process and therefore, is more appropriate to characterize and predict the osteophyte growth process than the deterministic models that only provide a certain value and neglect the person-to-person variation. Figure 4c demonstrates the osteophyte growth progression modeled by the stochastic model—the Wiener process. The ORIs at a fixed time instant are normally distributed and the distribution of the ORI keeps evolving over time. The black-dashed line represents the most probable ORI growth path. When the pain threshold is 0.15 (indicated with the red-solid horizontal line in Fig. 4c, the value is for illustrative purposes), the corresponding survival probability (i.e., survival function), which is the probability that the ORI is smaller than the pain threshold, keeps decreasing over time, as shown in Fig. 4d–i. The distributions of the ORI in the future can be obtained based on the Wiener process. The medical professionals can provide more reasonable advice regarding the possible consequences and the corresponding probabilities given the current situation. To the best of the authors' knowledge, it is relatively new to apply stochastic models to predict osteophyte growth progression and obtain evaluation metrics that can provide valuable information for clinical decision-making.

Some limitations should be noted in our study. Firstly, the dataset includes the osteophyte formation and growth data of twenty-three cervical vertebrae and seventy-four lumbar vertebrae. Although the sample size is enough for parameter estimation and model validation, a larger quantity of data should be enrolled in future studies for more robust validation and more accurate estimation of the general population parameters. Secondly, this study proposes a basic model where the osteophyte formation and growth process are considered independent and the growth process is homogeneous. However, other behaviors, such as disc height reduction^38^, may occur simultaneously or successively with the osteophytes during the cervical and lumbar vertebrae degeneration and are reported to affect the osteophyte formation time and growth rate^39^. In addition, Gelse et al.^40^ proposed that osteophyte growth shows a multi-stage pattern on the cell biological level. Therefore, both its association with other degenerative behaviors and its multi-stage behavior could be quantitatively investigated and enrolled into the future model for a more precise description (e.g., a narrower confidence interval) of osteophyte formation and growth process.

## Methods

**Figure 5:**
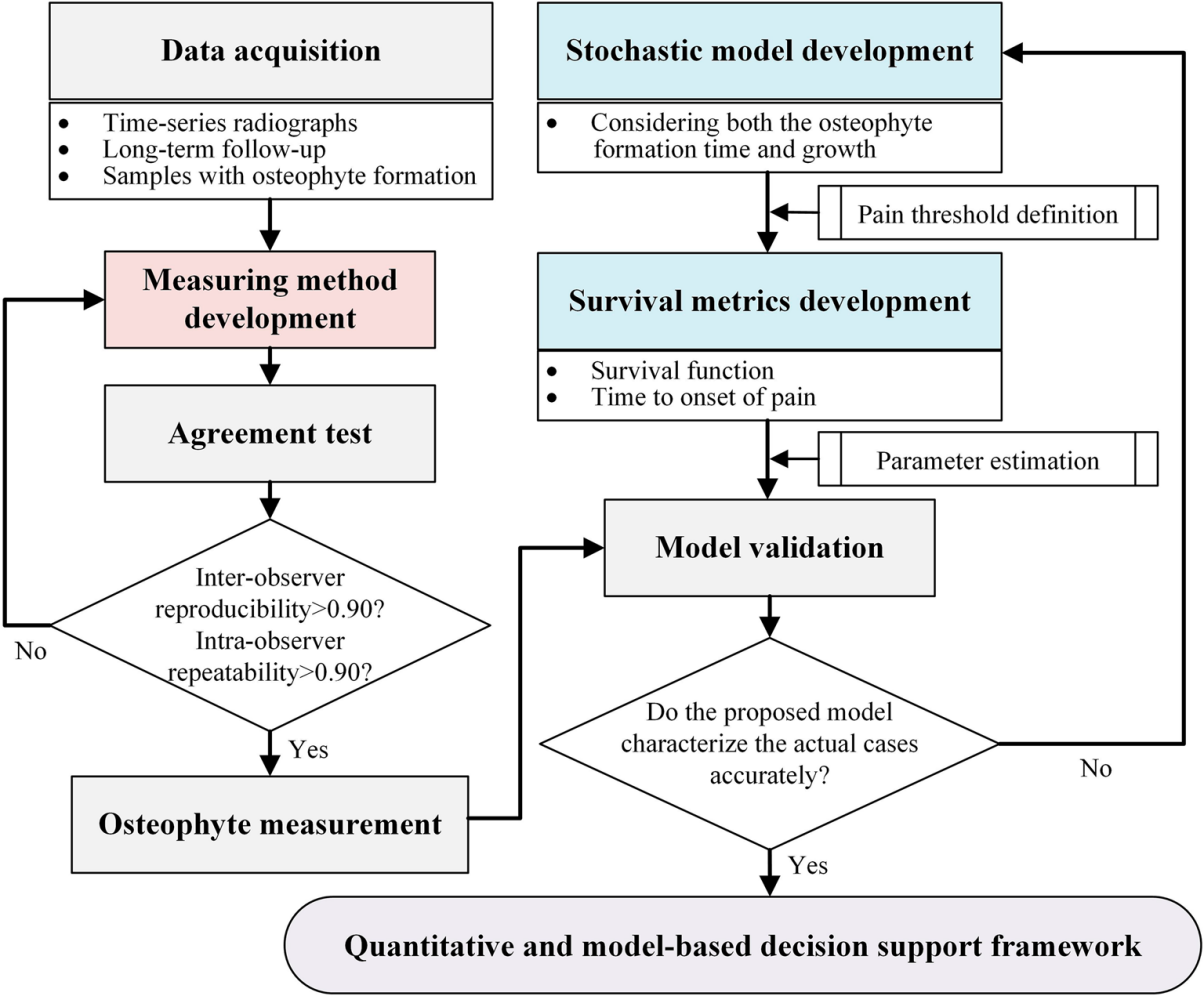
The workflow diagram.

Figure 5 shows the workflow of our study. We develop a measuring method for the anterior osteophyte length and conduct an agreement test to show the robustness of our method. In addition, we develop a stochastic model for characterizing the osteophyte formation and growth process and derive the related survival metrics for clinical application purposes. Time-series radiographic datasets are obtained from the hospital for model validation. Based on the robust measuring method and accurate models, we propose a quantitative and model-based decision support framework for treatment planning of cervical and lumbar osteophytes in clinical practices. The detailed information is found in the subsequent subsections.

### Dataset description

This study focuses on the osteophyte formation time and time-dependent osteophyte growth on both cervical and lumbar vertebrae. Radiographic osteophyte formation and growth data collected from September 2009 to September 2021 are obtained from West China Hospital, Sichuan University. As the osteophytes most commonly form on the five cervical vertebrae C3 through C7 and rarely on C1 and C2^41^, we focus on the five levels C3-C7 to investigate the osteophyte formation and growth behavior. For lumbar vertebrae, as osteophytes are frequently reported to form on vertebrae L1 through L5^42^, the five levels are included in the dataset for lumbar osteophyte investigation. Males and females are reported to show similar patterns in osteophyte development^43^, so the gender is not considered in our study.

**Figure 6:**
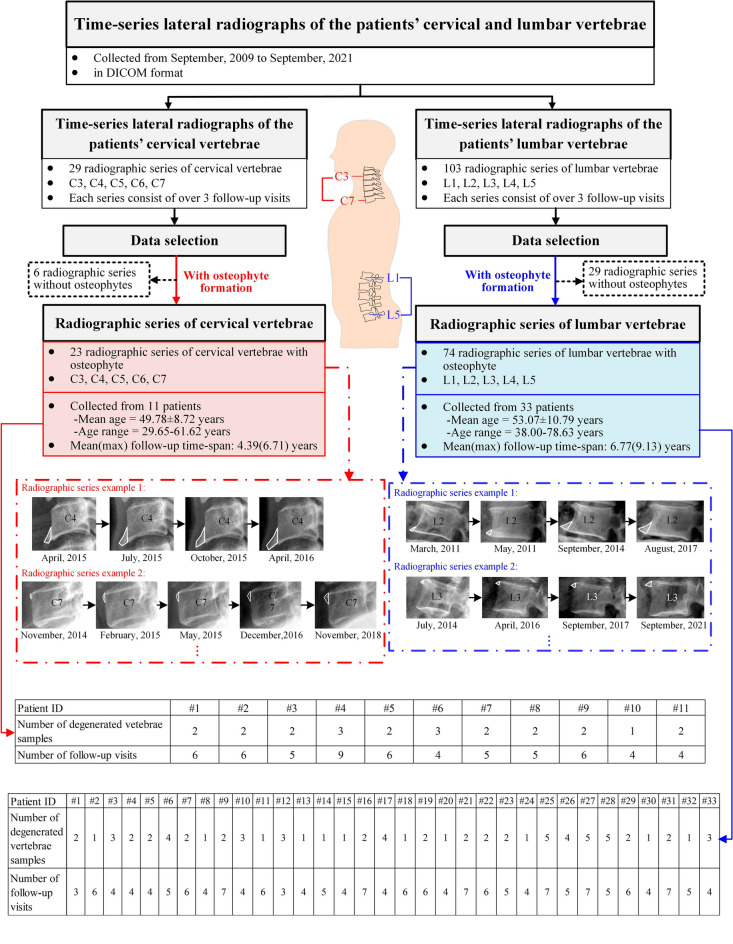
Database organization diagram.

Figure 6 shows the database organization. Our dataset includes 29 radiographic series of cervical vertebrae from C3 to C7 (23 with osteophytes and 6 without osteophytes) and 103 radiographic series of lumbar vertebrae from L1 to L5 (74 with osteophytes and 29 without osteophytes), where each series corresponds to a vertebra and is collected during the patient’s follow-up visits as shown in Fig. 6. The radiographic series of cervical and lumbar vertebra osteophytes are collected from 11 (at their first visit: mean age = 49.78 ± 8.72 years, age range = 29.65–61.62 years) and 33 (at their first visit: mean age = 53.07 ± 10.79 years, age range = 38.00–78.63 years) patients, respectively. One radiograph is taken for the vertebra and shows the osteophyte growth level of the vertebra during each visit. To protect the privacy of the patients, the specific dates of the radiographic images are not shown in Fig. 6. The mean(max) follow-up time-span of patients with cervical and lumbar osteophytes are 4.39(6.71) and 6.77(9.13) years, respectively. Each series consists of over 3 follow-up visits. Radiographs are of high resolution and are viewed by RadiAnt DICOM Viewer.

### Measurement of anterior osteophyte length based on lateral radiographs

#### Measuring methods

In this study, a new indicator Osteophyte Ratio Index (ORI) is proposed as follows:

1 $$ORI = \frac{{h_{1} }}{{h_{0} }}$$

where $h_{1}$ denotes the length of the osteophyte and $h_{0}$ denotes the width of the cervical or lumbar vertebra body in the lateral view of the radiographic image.

**Figure 7:**
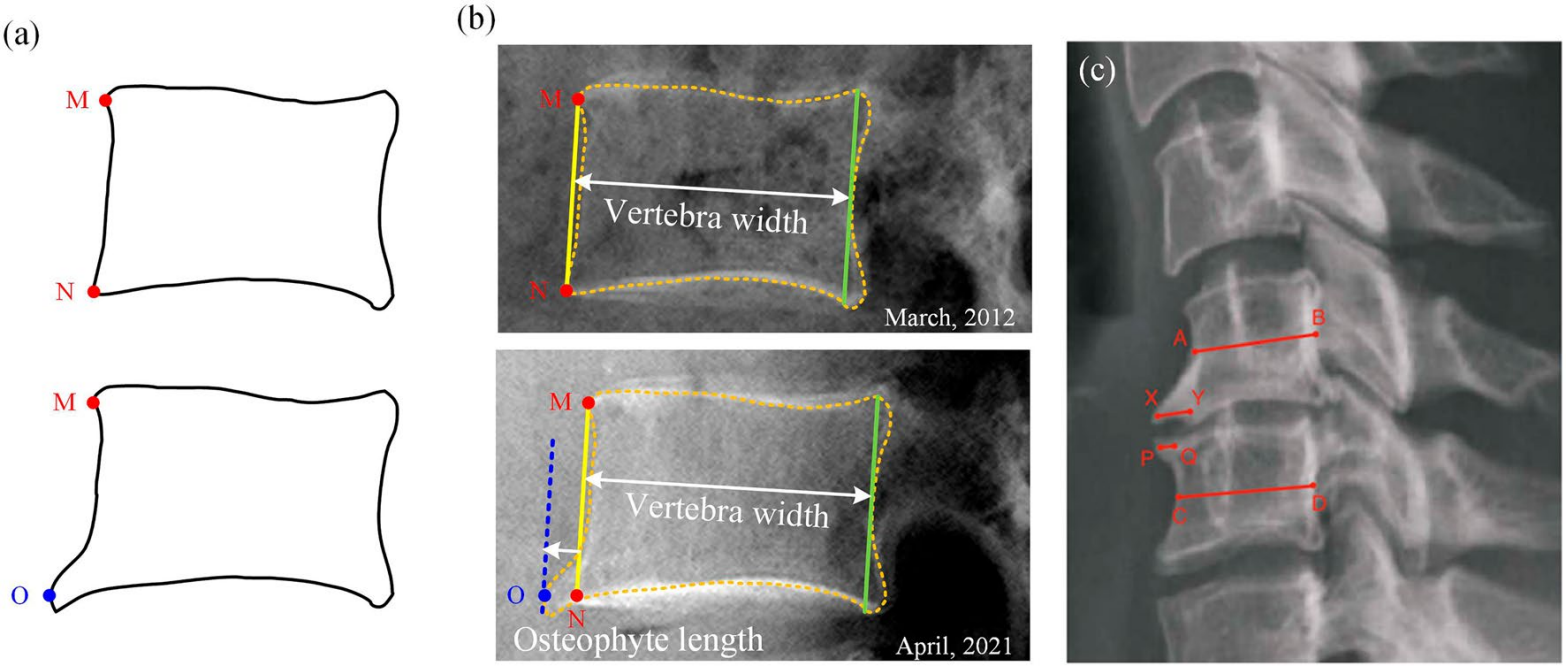
(**a**) A normal vertebra (the top one) and a degenerated vertebra (the bottom one) with osteophyte in the anterior side (lower left-hand corner). Points M and N are the anterior edge points of the normal vertebra in the lateral view. Point O is the osteophyte tip in the lateral view. (**b**) Measurement of osteophyte length and vertebra width. An orange dashed line is drawn along the vertebra contour for illustrative purposes. The measurement method includes three steps. First, the points M and N are identified and a yellow line connecting the two points is drawn. A second line (green line) is drawn parallel to the yellow line and tangential to the innermost part of the right vertebra contour. The distance between the yellow and green lines is defined as the vertebra width (white double-sided arrow). A third line (blue dashed line) is drawn parallel to the yellow line and tangential to the osteophyte tip. The distance between the yellow and blue lines is defined as the osteophyte length (white single-sided arrow). (**c**) Measurement of osteophyte length in Walraevens et al.^12^’s study. The osteophyte length (XY and PQ) is measured with respect to the anteroposterior diameter of the vertebral body (AB and CD, respectively). AB and CD are measured at the middle of the vertebral body.

The detailed measuring steps are in the Fig. 7 legend. The measurement is conducted with the RadiAnt DICOM Viewer^44^.

### Agreement test

Inter-observer reproducibility (i.e., the agreement between the measurements of two observers) and intra-observer repeatability (i.e., the agreement between the measurements of the same observer) of ORI are evaluated using the intra-class correlation coefficient (ICC) and percentage difference (PD)^45^. According to^46^, four observers (observers I, II, III and IV) are included to measure the lengths of anterior osteophytes using ORI independently from a subset of 50 randomly selected radiographs. Observers I, II, and III are a 35-year-old radiologic technologist, a 30-year-old orthopedist and a 23-year-old graduate student who majors in medical imaging technology, respectively. Observer IV is a 20-year-old undergraduate student who majors in engineering and has no clinical background. Each observer measures two rounds at a 2-week interval. The interpretation of ICC is based on the previous study^23^ where ICC ≤ 0.50, 0.50 < ICC ≤ 0.75, 0.75 < ICC ≤ 0.90 and 0.90 < ICC refers to the poor, moderate, good and excellent agreement, respectively. It is reported that a smaller PD value indicates a lower difference and thus higher agreement^24^. For comparison, the same agreement test is performed by the same four observers on the index proposed in Walraevens et al.^12^’s study.

### Stochastic model development

The assumptions of the osteophyte formation and growth process of a single cervical/lumbar vertebra are made as follows: (1) After a random time, osteophyte forms on one vertebra due to factors such as aging and mechanical stresses; (2) The osteophyte continues to grow after the formation following a stochastic process; (3) Only the largest osteophyte at each vertebra is investigated. (4) The pain occurs when the length of its anterior osteophyte reaches the pain threshold. (5) As the osteophytes on cervical and lumbar spinal vertebrae show similar etiology and growth patterns^47^, their formation and growth are assumed to follow the same stochastic process but with different parameters. The cervical vertebra at different levels (i.e., C3 through C7), are similar in shape and function^48,49^ and are treated as identical cervical subjects with the same parameters in our model. Likewise, lumbar vertebrae L1 to L5 are considered as identical lumbar subjects with the same parameters in our model as the degenerative behaviors are similar among the lumbar levels (i.e., L1 through L5)^50^.

**Figure 8:**
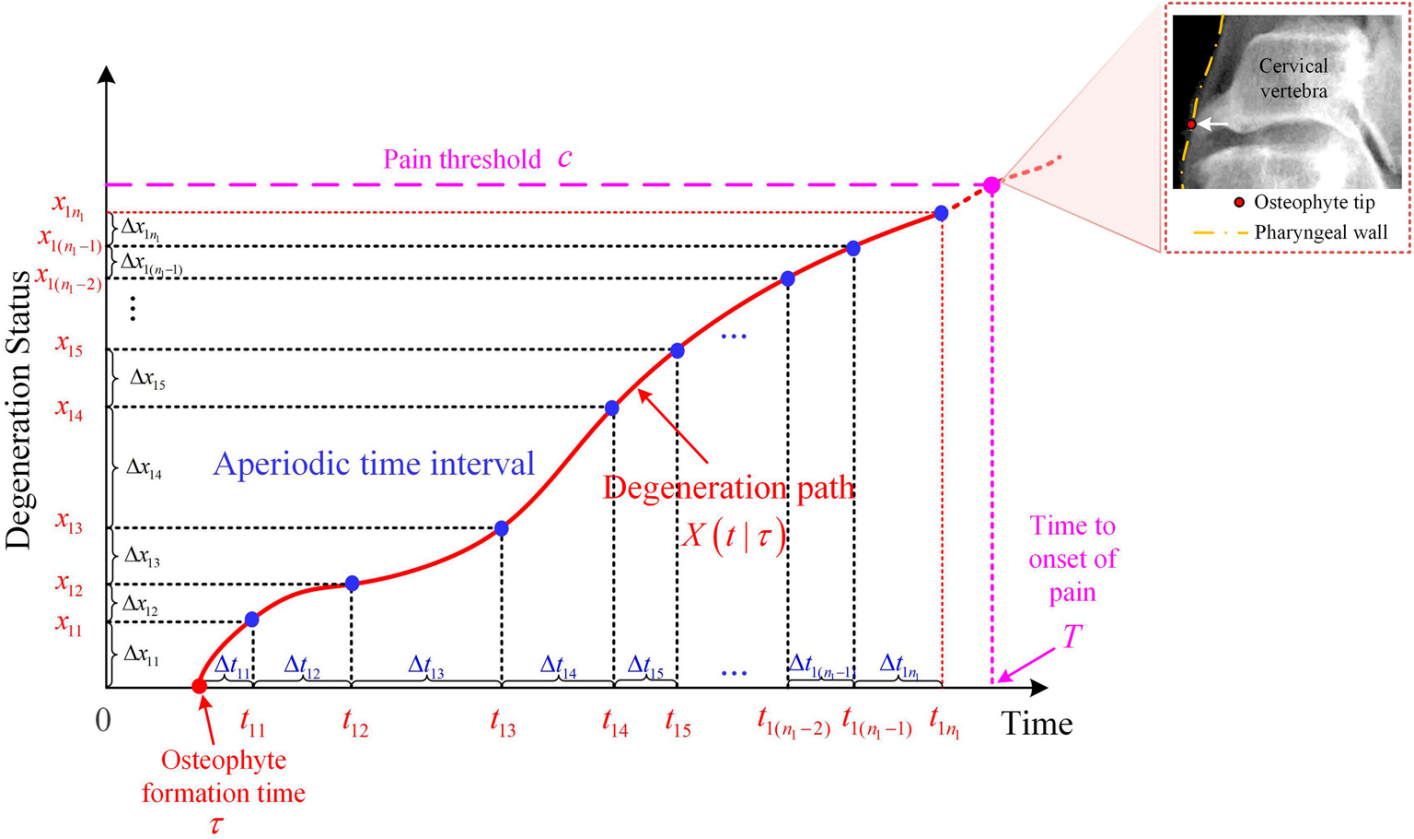
The schematic diagram of the osteophyte formation and growth on a single vertebra and the osteophyte growth data with aperiodic time interval.

The schematic diagram of the osteophyte formation and growth process is shown in Fig. 8. Let $\tau$ denote the osteophyte formation time of a vertebra. The degeneration status (i.e., osteophyte size) of the vertebra at time $t$ is denoted as $X\left( {t|\tau } \right)$. Note that $t - \tau$ is the length of time of its degeneration. The pain threshold $c$ corresponds to the maximum acceptable osteophyte size of the vertebra and $T$ corresponds to the time to onset of pain (TTOOP) when the degeneration status reaches $c$. Take the cervical vertebra as an example, pain occurs when a large anterior osteophyte compresses the pharyngeal wall (as illustrated in Fig. 8).

Note that $\tau$ is a random variable that reflects the biological properties of the cervical and lumbar vertebrae influenced by environmental and biological factors, physical activities, etc. In biomedical survival analysis, the two-parameter Weibull distribution is widely used to describe the time to develop a disease and is flexible in characterizing age-related diseases^51^. In this study, the osteophyte formation time since the patients' birthdays (i.e., age in years) is modeled with a Weibull distribution^52^.

In previous studies, a positive correlation has been found between the osteophyte index and age based on the linear regression models^13^. As it is observed that the osteophyte growth process has a linear trend and involves uncertainties, the widely implemented Wiener process^53^ is suitable for characterizing the osteophyte growth path. Let $X\left( {t|\tau } \right)$ denote the conditional osteophyte growth status of a cervical or lumbar vertebra at time $t$, given its osteophyte formation time $\tau$. For $\forall t > \tau$, $X\left( {t|\tau } \right)$ follows a normal distribution $N\left( {\mu \left( {t - \tau } \right),\sigma^{2} \left( {t - \tau } \right)} \right)$.

In an osteophyte formation and growth process, the unconditional probability density function (PDF) of the degeneration status of a cervical or lumbar vertebra at time $t$ is given by Eq. (2):

2 $$\begin{aligned} h\left( {x|t} \right) = & \int\limits_{0}^{t} {f\left( \tau \right)h\left( {x|t,\tau } \right)d\tau } \\ & \quad = \int\limits_{0}^{t} {\frac{b}{{a^{b} }}\tau^{{\left( {b - 1} \right)}} \exp \left\{ { - \left( {\frac{\tau }{a}} \right)^{b} } \right\}\frac{1}{{\sigma \sqrt {t - \tau } \sqrt {2\pi } }}\exp\left\{{{ - \frac{{\left[ {x - \mu \left( {t - \tau } \right)} \right]^{2} }}{{2\sigma^{2} \left( {t - \tau } \right)}}}}\right\} d\tau } \\ \end{aligned}$$

where $a > 0$ and $b > 0$ are the scale and shape parameters of the Weibull distribution. $x$ is the degeneration status of the cervical or lumbar vertebra, the drift parameter $\mu$ and the diffusion parameter $\sigma$ correspond to the mean growth rate and the volatility of the osteophyte’s growth respectively.

### Pain threshold definition

The anterior osteophyte that grows continuously on the cervical/lumbar vertebra may mechanically compress spinal nerve roots or soft tissue structures and lead to neck/back pain ^8,9^. The pain threshold refers to the maximum clinically-acceptable osteophyte size. We assume that the patient with an osteophyte on cervical/lumbar vertebra that has an ORI value exceeding the pain threshold will experience neck/back pain. Given the pain threshold value $c$, the statistics including the time to onset of pain (TTOOP) distribution, survival function and mean time to onset of pain (MTTOOP) are derived based on the developed model. These statistics predict when the pain will occur and imply the probability that the patient will survive without pain. They can provide references for early intervention before the pain actually occurs.

In clinical practice, the pain thresholds are determined as 0.52 and 0.22 for cervical and lumbar vertebrae, respectively. The determination of the pain thresholds are as follows. According to the literature^54^, the anterior cervical osteophyte with an average length of over 10 mm will cause mechanical compression on the neck. Since the mean anteroposterior (AP) diameter of the cervical vertebral body is 19.13 mm^55^, we recommend the pain threshold $c_{c} = 10/19.13 = 0.52$ for the cervical vertebra. Likewise, as Kojima et al.^56^ propose that the anterior lumbar osteophyte length greater than 10 mm is associated with low back pain and the mean AP diameter of the lumbar vertebral body is 46.31 mm^57^, we recommend the pain threshold $c_{l} = 10/46.31 = 0.22$ for lumbar vertebra. Note that the 0.52 and 0.22 are population-based recommended values, which are flexible and can be modified accordingly based on patient-specific characteristics. For instance, software or clinicians can measure the patient-specific AP diameter of the cervical/lumbar vertebral body on the patient’s lateral radiograph. The patient-specific pain threshold can be calculated by dividing the literature-recommended osteophyte length of 10 mm by the patient’s AP diameter.

### Survival metrics development

Note that as the osteophyte growth follows a Wiener process with drift $\mu$ and diffusion $\sigma$, TTOOP under pain threshold $c$ after its formation follows an IG distribution $\left( {T - \tau } \right) \sim IG\left( {\frac{c}{\mu },\frac{{c^{2} }}{{\sigma^{2} }}} \right)$ with a mean of $\frac{c}{\mu }$. Accordingly, the MTTOOP given its osteophyte formation time $\tau$ is $\frac{c}{\mu } + \tau$.

The PDF of TTOOP given the pain threshold $c$ is obtained as shown in Eq. (3):

3 $$\begin{aligned} g\left( {t|c} \right) = & \int\limits_{0}^{t} {f\left( \tau \right)g\left( {t|\tau ,c} \right)d\tau } \\ & \quad = \int\limits_{0}^{t} {\frac{b}{{a^{b} }}\tau^{{\left( {b - 1} \right)}} \exp \left\{ { - \left( {\frac{\tau }{a}} \right)^{b} } \right\}\sqrt {\frac{{c^{2} }}{{2\pi \sigma^{2} \left( {t - \tau } \right)^{3} }}} \exp \left\{ {\frac{{ - c^{2} \left( {t - \tau - \frac{c}{\mu }} \right)}}{{2\sigma^{2} \left( {\frac{c}{\mu }} \right)^{2} \left( {t - \tau } \right)}}} \right\}d\tau } \\ \end{aligned}$$

where $g\left( {t|\tau ,c} \right)$ denotes the conditional PDF of TTOOP of a cervical or lumbar vertebra given $\tau$ is known.

The survival function and MTTOOP for a given threshold $c$ are obtained in Eqs. (4) and (5):

4 $$\begin{aligned} R\left( {t|c} \right) = & \int\limits_{0}^{\infty } {H\left( {c|t,\tau } \right)f\left( \tau \right)d\tau } \\ & \quad = \int\limits_{0}^{t} {H\left( {c|t,\tau } \right)f\left( \tau \right)d\tau } + \left( {1 - F\left( t \right)} \right) \\ \end{aligned}$$

5 $$\begin{aligned} MTTF = & \int\limits_{0}^{\infty } {E\left( {T|\tau ,c} \right)} f\left( \tau \right)d\tau \\ & \; = \int\limits_{0}^{\infty } {\left( {\frac{c}{\mu } + \tau } \right)} f\left( \tau \right)d\tau \\ & \;\; = \frac{c}{\mu } + a\Gamma \left( {1 + \frac{1}{b}} \right) \\ \end{aligned}$$

where $H\left( { \cdot |t,\tau } \right)$ denotes the conditional survival function of a cervical or lumbar vertebra at time $t$ given $\tau$ is known, $\tau$ is the osteopyte formation time, $E\left( {T|\tau ,c} \right)$ denotes the conditional TTOOP of a cervical or lumbar vertebra given $\tau$ and $c$ are known.

The codes for the calculation of Eqs. (2), (3), (4) and (5) are provided in Supplementary A for clinical application purposes. Clinicians can obtain the corresponding plots by running the program on the MATLAB Platform. In addition, the program can provide personalized prediction and survival analysis based on adjusted versions of the equations. The MATLAB codes are provided in Supplementary B.

### Parameters estimation

The maximum likelihood estimation (MLE) algorithm is used to estimate the parameters of the model. The parameters of the proposed model include $\left\{ {a,b,\mu ,\sigma } \right\}$, where $a,b$ are the parameters of the distribution of the osteophyte formation time, and $\mu ,\sigma$ are the parameters that govern the osteophyte growth since the formation. It is assumed that the osteophyte formation and growth are independent and the likelihood functions for $\left\{ {a,b} \right\}$ and $\left\{ {\mu ,\sigma } \right\}$ are obtained independently. Parameters of the proposed model for cervical and lumbar vertebrae are estimated by the same method from separate data sources.

### Parameters estimation of the distribution of osteophyte formation time

Assume the radiographic image series of a total of $M$ cervical vertebrae are available. Among them, $N$ vertebrae are found to have developed osteophyte over the data-collection period. Let $\tau_{i}$, $1 \le i \le N$ denote the osteophyte formation time of the osteophyte on the $i^{th}$ cervical vertebra, $\tau_{k}$, $N + 1 \le k \le M$ denote the right-censoring time where the osteophyte has not developed on the cervical vertebra by the time of the last follow-up visit.

Taking the natural logarithm of the joint density:

6 $$\begin{aligned} \ell \left( {a,b|{{\varvec{\uptau}}}} \right) = & \log \left[ {\left( {\frac{b}{{a^{b} }}} \right)^{N} \prod\limits_{i = 1}^{N} {\tau_{i}^{{\left( {b - 1} \right)}} } \times \exp \left\{ { - \sum\limits_{i = 1}^{N} {\left( {\frac{{\tau_{i} }}{a}} \right)^{b} } } \right\} \times \prod\limits_{k = N + 1}^{M} {\exp \left\{ { - \left( {\frac{{\tau_{k} }}{a}} \right)^{b} } \right\}} } \right] \\ & \; = N\log b - bN\log \left( a \right) + \left( {b - 1} \right)\sum\limits_{i = 1}^{N} {\log \left( {\tau_{i} } \right)} - \sum\limits_{i = 1}^{N} {\left( {\frac{{\tau_{i} }}{a}} \right)^{b} } - \sum\limits_{k = N + 1}^{M} {\left( {\frac{{\tau_{k} }}{a}} \right)^{b} } \\ \end{aligned}$$

The MLE estimators of $a$ and $b$ for the Weibull distribution are obtained by maximizing Eq. (6), which can be achieved by using the maxLik package in R.

As the actual radiographic images are not obtained continuously, the exact osteophyte formation time (i.e., the time when ORI begins to exceed zero) is not observed directly. In this study, 23 cervical vertebrae and 74 lumbar vertebrae developed osteophytes over the follow-up period. Since 6 cervical and 29 lumbar vertebrae have not developed osteophytes, the corresponding patient’s age at the last follow-up date is denoted as the right-censored osteophyte formation time^58^. As the overall osteophyte growth process has a linear trend^13^, the actual osteophyte formation time of each cervical/lumbar vertebra is estimated by linear interpolation based on its first and last observations. Both the actual and the right-censored osteophyte formation time are the INPUT for parameter estimation of the Weibull distribution.

### Parameters estimation of the osteophyte growth process

The drift $\mu$ and diffusion $\sigma$ of the Wiener process are estimated as follows. Assume that a total of $N$ cervical vertebrae with osteophyte growing on them are available. There are $n_{i}$ measurements of osteophyte growth increment on the $i^{th}$ cervical vertebra. Let $\Delta x_{ij} \left( {1 \le i \le N,1 \le j \le n_{i} } \right)$ denote the $j^{th}$ osteophyte growth increment on the $i^{th}$ cervical vertebra and $\Delta t_{ij} \left( {1 \le i \le N,1 \le j \le n_{i} } \right)$ denote the corresponding time interval for osteophyte growth.

Note that in reality, although periodic follow-up visits are suggested for patients with osteophyte growth on their cervical vertebrae, the follow-up visits are usually aperiodic. Accordingly, the obtained osteophyte growth observations are also aperiodic, as illustrated in Fig. 8. The corresponding maximum likelihood estimators are obtained as follows.

The likelihood function of $\mu$ and $\sigma$ is:

7 $$L\left( {\mu ,\sigma |\Delta {\mathbf{x}},\Delta {\mathbf{t}}} \right) = \prod\limits_{i = 1}^{N} {\prod\limits_{j = 1}^{{n_{i} }} {\frac{1}{{\sigma \sqrt {\Delta t_{ij} } \sqrt {2\pi } }}\exp\left\{{{ - \frac{{\left[ {\Delta x_{ij} - \mu \Delta t_{ij} } \right]^{2} }}{{2\sigma^{2} \Delta t_{ij} }}}}\right\} } }$$

where $\Delta {\mathbf{x}}$ and $\Delta {\mathbf{t}}$ are the vectors of the degeneration increments and time increments. The MLE estimators $\left\{ {\hat{\mu },\hat{\sigma }} \right\}$ are obtained by maximizing the log-likelihood function using the maxLik package in R.

For the actual radiographic image datasets, all vertebrae samples with osteophytes developed are used for parameters estimation of the Wiener process. We censor the osteophyte growth data at time $t = 4$ for cervical vertebrae and $t = 6$ for lumbar vertebrae and use the data before the censor time for parameters estimation using MLE.

#### Ethical approval and consent to participate

This study was approved by the Institutional Review Board of West China Hospital. Written informed consent was obtained from all individual participants included in this study. We confirm that all methods were performed in accordance with the relevant guidelines and regulations.

### Supplementary Information

Supplementary Information.

## Data Availability

Summarized data have been presented and shared in this manuscript. The raw data that support the findings of this study are available from the West China Hospital but restrictions apply to the availability of these data, which were used under license for the current study, and so are not publicly available. Data are however available from the authors upon reasonable request and with permission of West China Hospital. Contact changxi.wang@scu.edu.cn to request the data from this study.
