# Supplementary material for: Quantitative analysis and stochastic modeling of osteophyte formation and growth process on human vertebrae based on radiographs: a follow-up study
Source: Sci Rep. 2024 Apr 24;14:9393. doi: 10.1038/s41598-024-60212-5 (PMC11043460; doi:10.1038/s41598-024-60212-5)
Supplement: Supplementary file 1 — Supplementary Information. [file 41598_2024_60212_MOESM1_ESM.pdf]

Quantitative Analysis and Stochastic Modeling of Osteophyte Formation and Growth Process on Human Vertebrae Based on Radiographs: A Follow-up Study

Tong Wu[1], **Changxi Wang**[1,3,*], **Kang Li**[1,2,3,*]

[1]West China Biomedical Big Data Center, West China Hospital, Sichuan University, Chengdu, 610041, China

[2]Orthopedics Department of West China Hospital, Sichuan University, Chengdu, 610041, China

[3]Sichuan University - Pittsburgh Institute, Sichuan University, Chengdu, 610207, China

*Corresponding: changxi.wang@scu.edu.cn, likang@wchscu.cn

Supplementary A: MATLAB Codes for Population-level Prediction and Survival Metrics

In this paper, the developed mathematic equations are mainly illustrated by plots as shown in Figures 1 and 2 for clinical use. As the pain thresholds used to obtain Figures 1 and 2 are for validation purposes and are different from 0.52 and 0.22 which we recommend for clinical application, the codes of our mathematic functions are needed for clinicians to obtain prediction plots under a specific pain threshold. Clinicians can obtain (i) the mean path and 95% CI plots as shown in Figures 1(d) and 2(d), (ii) the survival curve as shown in Figures 1(e) and 2(e), (iii) the PDF plot of the time to onset of pain as shown in Figures 1(f) and 2(f) and (iv) the predicted value of mean time to onset of pain by simply putting the codes into the MATLAB Platform. The sentences behind the "%" symbol are instructions for choosing different values under different scenarios.

- **i. MATLAB Code for Deriving the Mean Path and 95% CI Plot**

```
clc
clear
a=52.5216; %It is 52.5216 for the cervical vertebra and 54.9779 for the lumbar vertebra.
b=5.7469; %It is 5.7469 for the cervical vertebra and 2.3443 for the lumbar vertebra
miu=0.0145553; %It is 0.0145553 for the cervical vertebra and 0.012836 for the lumbar vertebra.
sigma=0.0113259; %It is 0.0113259 for the cervical vertebra and 0.024040 for the lumbar vertebra.
n_x=2000;
x_min=-1;
x_max=2;
X1=linspace(x_min,x_max,n_x);
delta_X=(x_max-x_min)/(n_x);
n_t=71;
delta_t=5;
F_1=zeros((n_t-1)/delta_t+1,1);
F_2=zeros((n_t-1)/delta_t+1,1);
M=zeros((n_t-1)/delta_t+1,1);
n=1000;
for t=1:delta_t:n_t
P=rand(n,1);
tau1=P(:)*t;
f=zeros(n_x,1);
for j=1:1:n_x
    x=X1(j);
    g=wblpdf(tau1,a,b).*normpdf(x,miu*(t-tau1),sigma*((t-tau1).^0.5));
f(j)=mean(g)*t;
end

I_1=find(cumsum(f*delta_X) >= 0.025*sum(f*delta_X), 1, 'first');
I_2=find(cumsum(f*delta_X) >= 0.975*sum(f*delta_X), 1, 'first');
F_1((t-1)/delta_t+1)=X1(1,I_1);
F_2((t-1)/delta_t+1)=X1(1,I_2);
```

```
C_1=X1'.*f*delta_X;
M((t-1)/delta_t+1)=sum(C_1(:));
end

figure
a111=plot(1:delta_t:n_t,F_1,'k:','Linewidth',1.5);hold on
a222=plot(1:delta_t:n_t,M,'r:','Linewidth',1.5);hold on
plot(1:delta_t:n_t,F_2,'k:','Linewidth',1.5);hold on
xlabel('Age (years)','fontname', 'times new roman','fontSize',12)
ylabel('The ORI','fontname', 'times new roman','fontSize',12)
set(gca,'XLim',[0 70])
legend([a111,a222],'The 95% confidence interval','The mean degeneration path','fontname', 'times new roman','FontSize',12,'Location','northwest','Orientation','vertical','Box','on')
```

- **ii. MATLAB Code for Deriving the Survival Curve**

```
clc
clear
a=54.9779; %It is 52.5216 for the cervical vertebra and 54.9779 for the lumbar vertebra.
b=2.3443; %It is 5.7469 for the cervical vertebra and 2.3443 for the lumbar vertebra
mu=0.012836; %It is 0.0145553 for the cervical vertebra and 0.012836 for the lumbar vertebra.
sigma=0.024040; %It is 0.0113259 for the cervical vertebra and 0.024040 for the lumbar vertebra.
c=0.22;%the pain threshold value is 0.52 and 0.22 for cervical and lumbar vertebrae respectively.
n_1=10000
n_t=90
T_min=10
T_max=100
T=linspace(T_min,T_max,n_t);
R_theo=zeros(n_t,1);
for t=1:1:n_t
    P=rand(n_1,1);
    tau1=P(:)*t;
    g_1=wblpdf(tau1,a,b).*normcdf(c,mu*(t-tau1),sigma*(t-tau1).^0.5);
    R_theo(t)=mean(g_1)*t+1-wblcdf(t,a,b);
end
X=T'
figure
a1=plot(T,R_theo,'-b','Linewidth',1.2);hold on;
xlabel('Age (years)','fontname', 'times new roman','fontSize',12)
ylabel('Survival probability','fontname', 'times new roman','fontSize',12)
set(gca,'fontSize',12)
set(gca,'xtick',10:10:100)
legend('Theoretical survival curve', 'fontname','times new roman','FontSize',12,'Location','southwest','Orientation','vertical','Box','on')
```

- **iii. MATLAB Code for Deriving the PDF Plot of the Time to Onset of Pain**

```
clc
clear
a=54.9779; %It is 52.5216 for the cervical vertebra and 54.9779 for the lumbar vertebra.
b=2.3443; %It is 5.7469 for the cervical vertebra and 2.3443 for the lumbar vertebra
mu=0.012836; %It is 0.0145553 for the cervical vertebra and 0.012836 for the lumbar vertebra.
sigma=0.024040; %It is 0.0113259 for the cervical vertebra and 0.024040 for the lumbar vertebra.
c=0.22;%the pain threshold value is 0.52 and 0.22 for cervical and lumbar vertebrae respectively.
n_t=24;
t_min=10;
t_max=80;
T=linspace(t_min,t_max,n_t);
pd = makedist('InverseGaussian','mu',c/mu,'lambda',(c^2)/(sigma^2));
n=20000;
g=zeros(n_t,1);
for j=1:1:n_t
    t=T(j);
    P=rand(n,1);
    Tau1=P(:)*t;
    p=wblpdf(Tau1,a,b).*pdf(pd,(t-Tau1));
    g(j)=mean(p)*t;
end
figure
kk=plot(T,g,'-b','Linewidth',1.2);hold on
xlabel('Time to onset of pain (years)','fontname', 'times new roman','fontSize',12)
ylabel('Probability density','fontname', 'times new roman','fontSize',12)
legend('The estimated PDF', 'fontname','times new roman','FontSize',12,'Location','northwest','Orientation','vertical','Box','on')
```

- **iv. MATLAB Code for Deriving the Value of Mean Time to Onset of Pain**

```
clc
clear
a=54.9779; %It is 52.5216 for the cervical vertebra and 54.9779 for the lumbar vertebra.
b=2.3443; %It is 5.7469 for the cervical vertebra and 2.3443 for the lumbar vertebra
mu=0.012836; %It is 0.0145553 for the cervical vertebra and 0.012836 for the lumbar vertebra.
sigma=0.024040; %It is 0.0113259 for the cervical vertebra and 0.024040 for the lumbar vertebra.
c=0.22;%the pain threshold value is 0.52 and 0.22 for cervical and lumbar vertebrae respectively.
[M V]=wblstat(a,b);
MTTOOP_theo=c/mu+M
```

Supplementary B: MATLAB Codes for Patient-specific Prediction and Survival Metrics

Clinicians can obtain (i) the mean path and 95% CI plots, (ii) the survival curve and (iii) the PDF plot of

the time to onset of pain with its mean value as shown in Figures 3(b) by simply putting the codes into the MATLAB Platform. The sentences behind the "%" symbol are instructions for choosing different values under different scenarios.

- **i. MATLAB Code for Deriving the Mean Path and 95% CI Plot**

```
clc
clear
a=54.9779; %It is 52.5216 for the cervical vertebra and 54.9779 for the lumbar vertebra.
b=2.3443; %It is 5.7469 for the cervical vertebra and 2.3443 for the lumbar vertebra
miu=0.012836; %It is 0.0145553 for the cervical vertebra and 0.012836 for the lumbar vertebra.
sigma=0.024040; %It is 0.0113259 for the cervical vertebra and 0.024040 for the lumbar vertebra.
x0=0.15;%It is the measured ORI value of the patient's osteophyte.
tpre=10;%It is the years we want to predict.

cf111=zeros(tpre+1,1)
for t=0:1:tpre
   mu = t*miu;
   sig = sigma*(t)^0.5;
   cf111((t+1),:)=norminv([0.975],mu,sig)

end
cf111(find(isnan(cf111)==1)) = 0
cf111=cf111+x0;

cf333=zeros(tpre+1,1)
for t=0:1:tpre
      mu = t*miu;
      sig = sigma*(t)^0.5;
    cf333((t+1),:)=norminv([0.025],mu,sig)
end
cf333(find(isnan(cf333)==1)) = 0
cf333=cf333+x0;

figure
a111=plot(0:1:tpre,cf111,'b--','Linewidth',2);hold on
x=0:1:tpre;
m=miu*x+x0;
a222=plot(0:1:tpre,m,'r-','Linewidth',2);hold on
a333=plot(0:1:tpre,cf333,'b--','Linewidth',2);hold on
a444=scatter(0,x0,'ko','filled','LineWidth',10)
xlabel('Future timeline (years)','fontname', 'times new roman','fontSize',12)
ylabel('The ORI','fontname', 'times new roman','fontSize',12)
legend([a111,a222,a444],'The 95% confidence interval','The mean degeneration path','The current state','fontname', 'times new roman','FontSize',11,'Location','northwest','Orientation','vertical','Box','on')
```

- **ii. MATLAB Code for Deriving the Survival Curve**

```
clc
clear
a=54.9779; %It is 52.5216 for the cervical vertebra and 54.9779 for the lumbar vertebra.
b=2.3443; %It is 5.7469 for the cervical vertebra and 2.3443 for the lumbar vertebra
mu=0.012836; %It is 0.0145553 for the cervical vertebra and 0.012836 for the lumbar vertebra.
sigma=0.024040; %It is 0.0113259 for the cervical vertebra and 0.024040 for the lumbar vertebra.
x0=0.15;%It is the measured ORI value of the patient's osteophyte.
c=0.22;%the pain threshold value is 0.52 and 0.22 for cervical and lumbar vertebrae respectively.
%the pain threshold can also be modified based on the patient's personal characteristic
c0=c-x0;
tpre=10;%It is the years we want to predict.
R=zeros(tpre+1,1);
for t=0:1:tpre
    R(t+1,1)=normcdf(c0,mu*t,sigma*t^0.5)
end

figure
a1=plot(0:1:tpre,R,'-b','Linewidth',1.2);hold on;
a2=scatter(0,R(1),'ko','filled','LineWidth',10)
xlabel('Future timeline (years)','fontname', 'times new roman','fontSize',12)
ylabel('Survival probability','fontname', 'times new roman','fontSize',12)
set(gca,'fontSize',12)
legend([a1,a2],'Predicted survival curve','The current state', 'fontname','times new roman','FontSize',12,'Location','southwest','Orientation','vertical','Box','on')
```

- **iii. MATLAB Code for Deriving the PDF Plot and Mean Value of the Remaining Time to Onset of Pain**

```
clc
clear
mu=0.012836; %It is 0.0145553 for the cervical vertebra and 0.012836 for the lumbar vertebra.
sigma=0.024040; %It is 0.0113259 for the cervical vertebra and 0.024040 for the lumbar vertebra.
x0=0.15;%It is the measured ORI value of the patient's osteophyte.
c=0.22;%the pain threshold value is 0.52 and 0.22 for cervical and lumbar vertebrae respectively.
%the pain threshold can also be modified based on the patient's personal characteristic
c0=c-x0;
tpre=10;%It is the years we want to predict.
t=0:1:tpre;
f=(c0^2/2/pi./(t.^3)/(sigma^2)).^0.5.*exp(-((c0-mu*t).^2)/2./(sigma^2)./t);
MTTOOP=c0/mu
figure
a1=plot(t,f,'-b','Linewidth',1.2);hold on;
a2=scatter(5.4534,0,'ro','filled','LineWidth',20);
```

```
xlabel('Remaining time to onset of pain (years)','fontname', 'times new roman','fontSize',12)
ylabel('Probability density','fontname', 'times new roman','fontSize',12)
legend([a1,a2],'The estimated PDF','Mean remaining time to onset of pain', 'fontname','times new roman','FontSize',12,'Location','northeast','Orientation','vertical','Box','on')
```
